# A measles virus selectively blind to signaling lymphocytic activation molecule shows anti-tumor activity against lung cancer cells

**DOI:** 10.18632/oncotarget.4366

**Published:** 2015-06-30

**Authors:** Tomoko Fujiyuki, Misako Yoneda, Yosuke Amagai, Kunie Obayashi, Fusako Ikeda, Koichiro Shoji, Yoshinori Murakami, Hiroki Sato, Chieko Kai

**Affiliations:** ^1^ Laboratory Animal Research Center, The Institute of Medical Science, The University of Tokyo, Shirokanedai, Minato-ku, Tokyo, 108-8639, Japan; ^2^ Division of Molecular Pathology, The Institute of Medical Science, The University of Tokyo, Shirokanedai, Minato-ku, Tokyo, 108-8639, Japan

**Keywords:** measles virus, virotherapy, lung cancer, oncolytic virus, nectin-4

## Abstract

Lung cancer cells, particularly those of non-small-cell lung cancer, are known to express Nectin-4. We previously generated a recombinant measles virus that uses Nectin-4 as its receptor but cannot bind its original principal receptor, signaling lymphocyte activation molecule (SLAM). This virus (rMV-SLAMblind) infects and kills breast cancer cells *in vitro* and in a subcutaneous xenograft model. However, it has yet to be determined whether rMV-SLAMblind is effective against other cancer types and in other tumor models that more closely represent disease. In this study, we analyzed the anti-tumor activity of this virus towards lung cancer cells using a modified variant that encodes green fluorescent protein (rMV-EGFP-SLAMblind). We found that rMV-EGFP-SLAMblind efficiently infected nine, human, lung cancer cell lines, and its infection resulted in reduced cell viability of six cell lines. Administration of the virus into subcutaneous tumors of xenotransplanted mice suppressed tumor growth. In addition, rMV-EGFP-SLAMblind could target scattered tumor masses grown in the lungs of xenotransplanted mice. These results suggest that rMV-SLAMblind is oncolytic for lung cancer and that it represents a promising tool for the treatment of this disease.

## INTRODUCTION

Measles virus (MV) is a member of *Paramyxoviridae*. Since it was discovered that MV infects tumor cells and induces tumor regression [1], MV has been focused on as a candidate virotherapy tool for cancers.

MV uses three different molecules as its receptor to infect host cells; CD46 [2, 3], SLAM [4], and Nectin-4 [5, 6]. While MV vaccine strains use all three of these molecules, wild type MV strains use Nectin-4 and SLAM but not CD46 [7].

Nectin-4 is expressed in the human placenta, but only poorly in the tissues of other organs [8]. Recently, it has been reported that Nectin-4 is selectively up-regulated in a variety of tumor cell types including breast, ovarian, and lung cancer cells [9–12]. Therefore, Nectin-4 is a good target for these types of tumors, and thus, MV could be a good candidate tool to selectively attack tumor cells. In contrast, while CD46 is expressed more strongly in tumor cells than other cell types, it is expressed ubiquitously in all nucleated human cells [13, 14]. Current virotherapy using MV vaccine strains has focused on CD46 for ovarian cancer and myeloma [15, 16]. However, because CD46 is ubiquitously expressed, side effects and/or inefficient targeting to tumor cells remains to be considered.

SLAM is selectively expressed in immune cells that are the first targets of wild type MVs, causing profound immunosuppression in the host and promoting systemic viral spread in host [17]. Conversely, ablation of SLAM binding activity has been shown to attenuate the pathogenicity of the virus [18]. We previously developed a recombinant MV based on a wild-type HL strain that is blind to SLAM (rMV-SLAMblind), and demonstrated that infection with rMV-SLAMblind efficiently killed breast cancer cells *in vitro* and *in vivo* but lost MV pathogenicity when tested in monkey models [19]. To our knowledge, this was the first example to demonstrate that a wild type MV strain with mutations leading blindness to SLAM exerts anti-tumor activity. In addition, the anti-tumor activity of rMV-SLAMblind was higher than that of a MV vaccine strain [19]. We also demonstrated that the pathogenicity of rMV-SLAMblind was actually attenuated, because monkeys did not show any measles symptoms after subcutaneous inoculation [19]. Therefore, it is expected that rMV-SLAMblind, because it selectively targets tumor cells, is a good candidate as a tool for cancer therapy. However, the anti-tumor effects of rMV-SLAMblind were shown only in human breast cancer cell lines. Previous work has suggested that the Nectin-4 expression level varies among types of cancer, but the expression of this receptor has not been investigated comprehensively [11]. To understand the range of rMV-SLAMblind-applicable cancers, additional types of tumors need to be examined.

Lung cancer remains the most common cause of cancer death, and effective therapies are urgently needed. Recently, Nectin-4 (also called poliovirus receptor related-4/PVRL4) was identified as a possible diagnostic and therapeutic target for lung cancer, and may represent a better diagnostic biomarker for non-small-cell lung cancer (NSCLC) than other known markers with respect to sensitivity and specificity [12]. Lung cancer is divided into small cell lung cancer (SCLC) and NSCLC. Cell lines of NSCLC are generally less sensitive to radiation than SCLC cell lines [20] and over 70% of patients with NSCLC in late-stage do not respond to chemotherapy [21], which accounts for approximately 85% of all lung cancer cases [22].

In this study, we demonstrated that rMV-SLAMblind can infect and kill lung cancer cells, by targeting Nectin-4, in particular NSCLC cells, both *in vitro* and *in vivo* resulting in cell death and tumor regression.

## RESULTS

**Figure 1:**
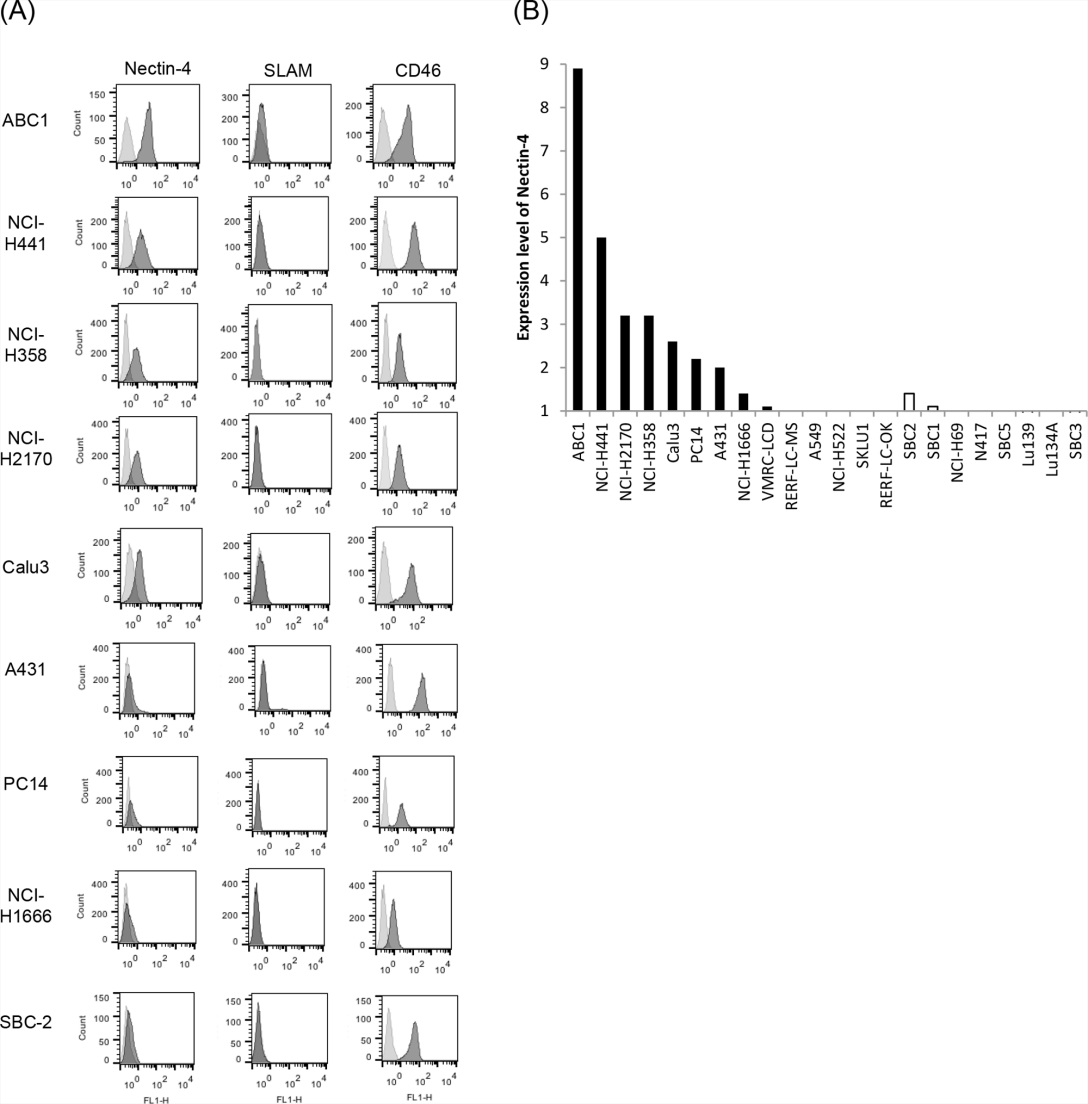
Expression of MV receptors on lung cancer cells **A.** The expression of MV receptor molecules on the surface of the cells from a panel of lung cancer cell lines was analyzed by flow cytometry (MV receptor; black histogram, isotype control; gray histogram). **B.** Receptor expression is presented as the ratio of the MFI for the receptor and isotype control antibody groups. The solid bar denotes NSCLC, and the open bar denotes SCLC.

**Figure 2:**
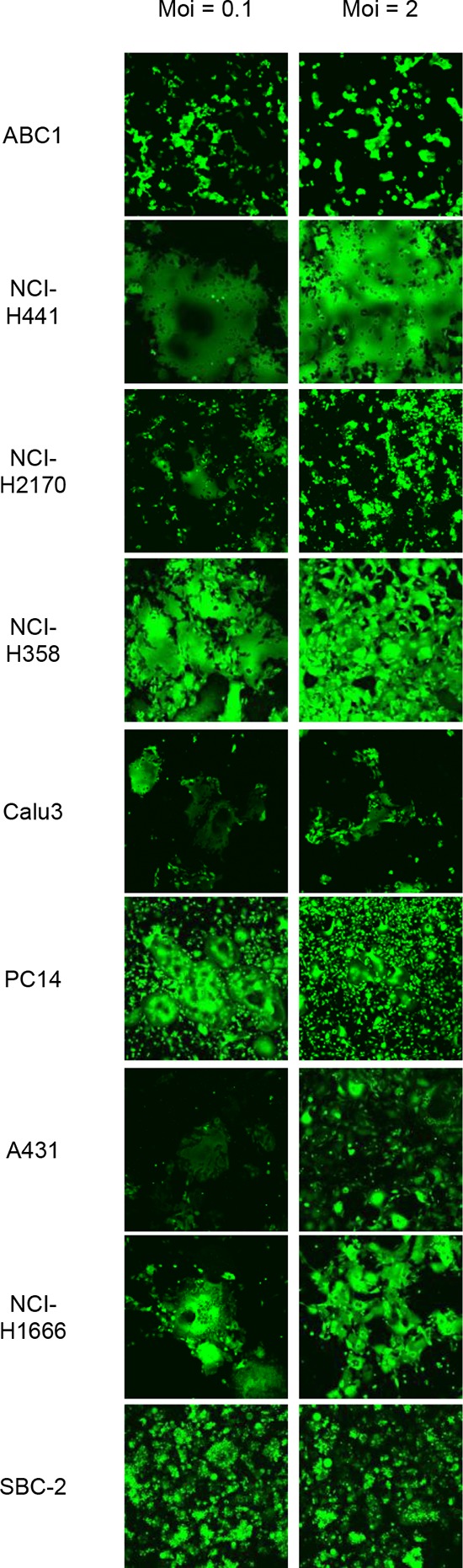
Susceptibility of Nectin-4 expressing-lung cancer cells to rMV-SLAMblind infection Representative images showing the efficiency of rMV-EGFP-SLAMblind infection in a panel of lung cancer cell lines. Cells were inoculated with rMV-EGFP-SLAMblind at a moi of 0.1 or 2. Maginification; ×100.

Flow cytometry was used to evaluate Nectin-4 expression in a panel of lung cancer cell lines, including 14 NSCLC lines and eight SCLC lines. Nectin-4 expression varied among the different cell lines analyzed and was clearly detected in eight of the 14 tested NSCLC cell lines (ABC1, NCI-H441, NCI-H2170, NCI-H358, Calu-3, PC14, A431, and NCI-H1666), and in one (SBC-2) of the eight tested SCLC cell lines (Figure 1). To examine whether other MV receptors were expressed on these cells, CD46 and SLAM expression were also analyzed. CD46 was expressed in all of the analyzed cell lines, whereas SLAM expression was barely detectable (Figure 1A). When cells were inoculated with rMV-EGFP-SLAMblind at a multiplicity of infection (moi) of 0.1 or 2, all Nectin-4-expressing cells became fluorescent and developed syncytia, a hallmark of MV infection (Figure 2).

**Figure 3:**
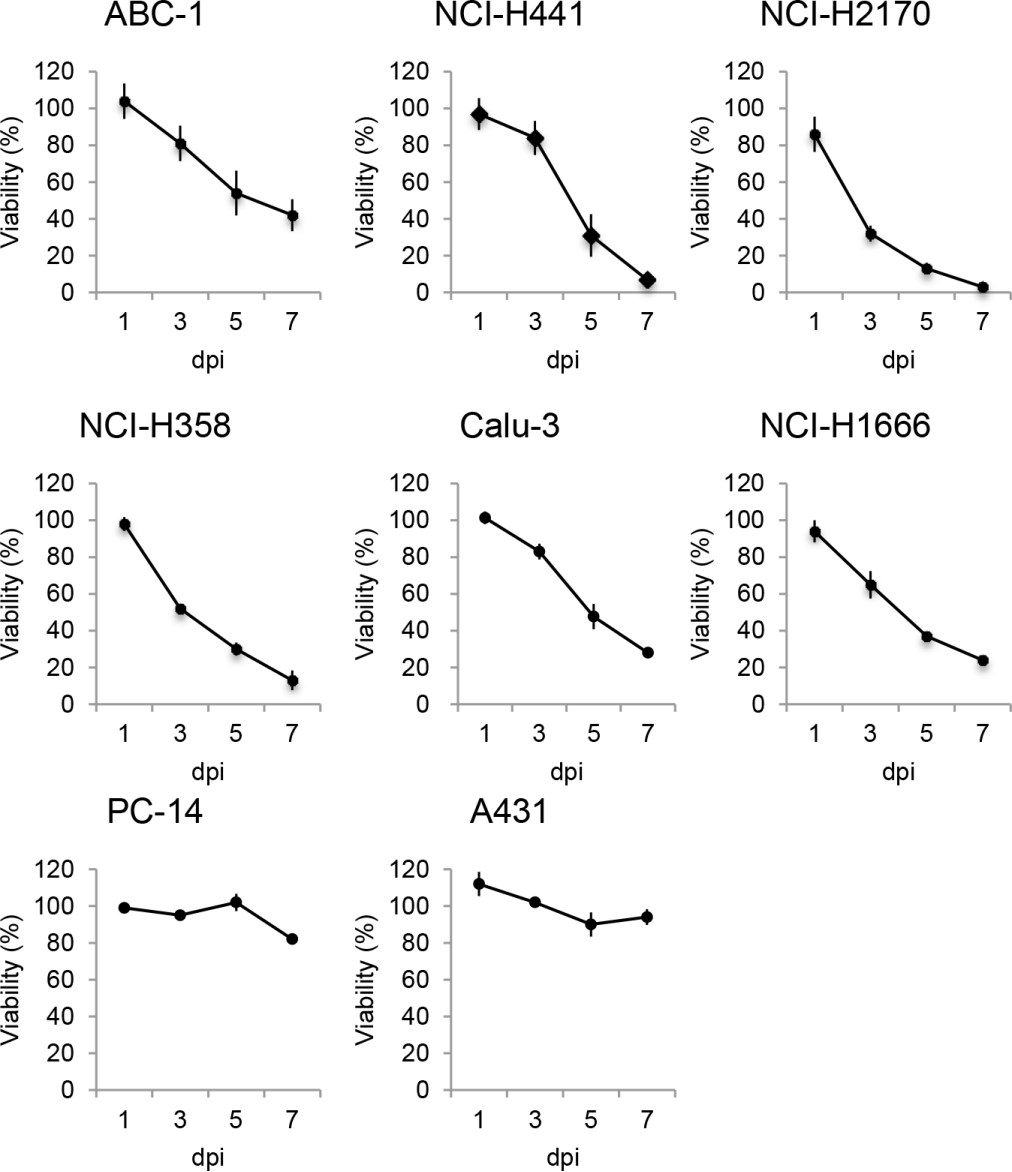
Oncolytic activity of rMV-SLAMblind towards lung cancer cells *in vitro* Cells were inoculated with rMV-EGFP-SLAMblind at a moi of 1. Cell viability was measured at 1, 3, 5, and 7 dpi by WST-1 assay. Data are presented as means ± SEM of three independent experiments.

To examine whether rMV-SLAMblind infection results in the death of the Nectin-4 expressing cells, viability assays were performed on the eight NSCLC cell lines (ABC1, NCI-H441, NCI-H2170, Calu-3, NCI-H358, PC14, A431, and NCI-H1666) after inoculation with rMV-EGFP-SLAMblind. By 7 dpi, the viabilities of ABC1, NCI-H441, NCI-H2170, NCI-H358, Calu-3, and NCI-H1666 cells decreased by more than 60% (Figure 3). Of the eight cell lines studied, ABC1, NCI-H441, H2170, H358, and Calu-3 exhibited relatively higher levels of Nectin-4 expression (Figure 1A), and cell death was observed for all of these cell lines following virus infection. NCI-H1666, PC14 and A431 cells exhibited lower level of Nectin-4 expression, and in the case of PC14 and A431, no obvious decrease in cell viability was observed, although a reduced viability was observed for NCI-H1666. These results suggest that the cytotoxic effect of rMV-EGFP-SLAMblind tends to correlate with the level of Nectin-4 expression in target cells.

**Figure 4:**
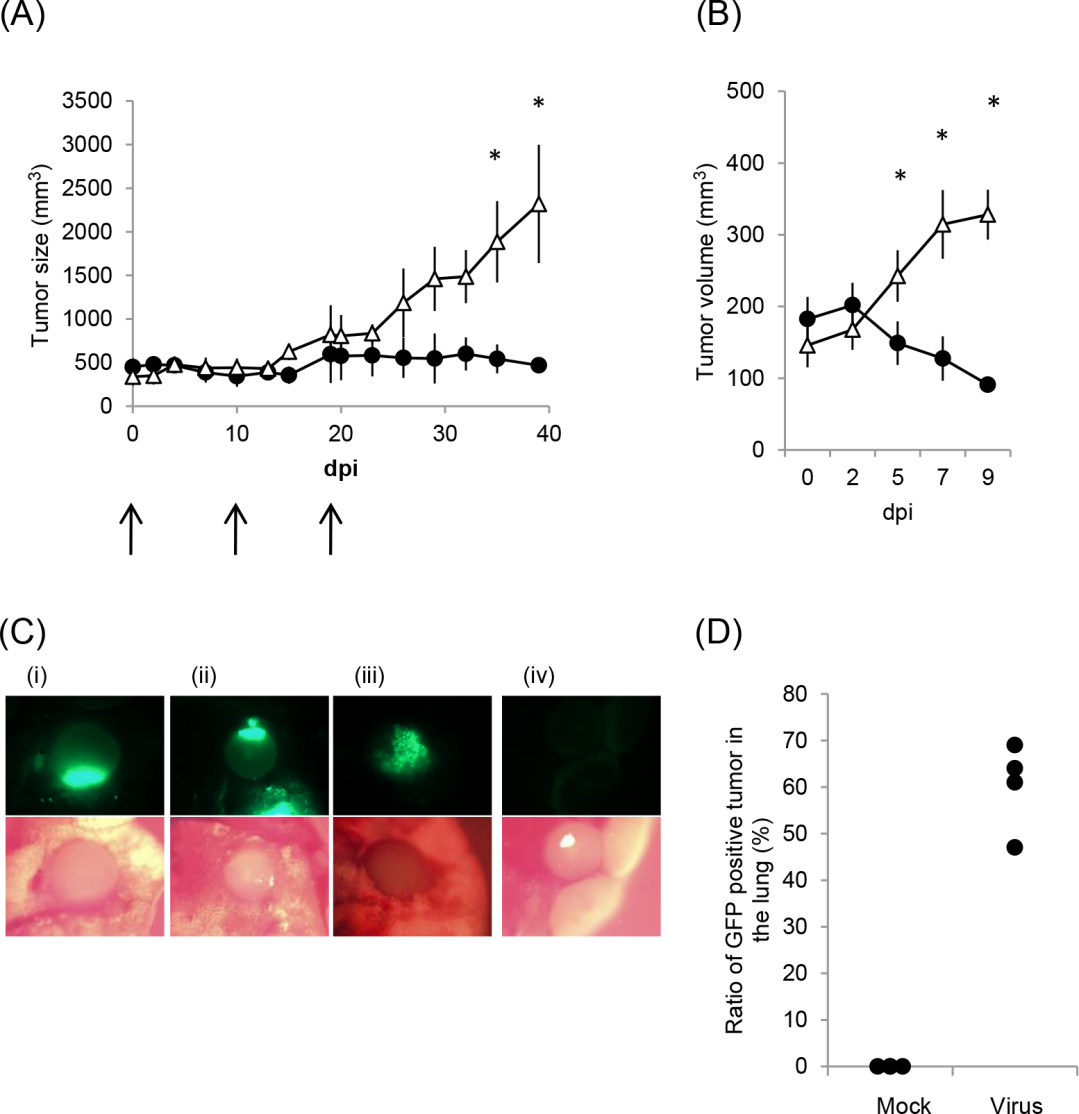
Anti-tumor effect of rMV-SLAMblind in a xenograft model of a lung cancer **A.** and **B.** The volume of tumors, derived from subcutaneous NCI-H441 xenografts, are shown following intratumoral administration of rMV-EGFP-SLAMblind. Data are presented as means ± SEM. * *p* < 0.05 (Wilcoxon Rank Sum test). (A) Virus (filled circle) or medium (open triangle) was administered three times at 0, 10, and 19 days post-first inoculation (*n* = 4). (B) Virus (filled circle) or medium (open triangle) was administered once (*n* = 8 for mock and *n* = 9 for virus administration). **C.** and **D.** NCI-H441/CMV-Luc cells were injected intravenously into mice and rMV-EGFP-SLAMblind subsequently serially administered intravenously after lung tumors had established. (C) The lung and tumors of the mice treated with the virus (i–iii) or medium (d) were observed by fluorescent microscopy. (D) The proportion of tumors in the lung exhibiting fluorescence is shown. Each dot indicates the data obtained from an individual mouse.

We next examined whether rMV-SLAMblind has an anti-tumor effect *in vivo* using a mouse xenograft model. NCI-H441 cells were selected for *in vivo* experiments, because of their high Nectin-4 expression, the cytotoxic effect of rMV-EGFP-SLAMblind towards these cells *in vitro*, and the proven use of this cell line in xenograft models in SCID mice [24]. NCI-H441 cells were transplanted subcutaneously into SCID mice. Once tumors had been established, 1 × 10^6^ TCID_50_ of rMV-EGFP-SLAMblind was administered three times intratumorally. Administration of the virus resulted in a suppression of tumor growth when compared with control treatment (Figure 4A). When virotherapy was started for mice bearing smaller tumors, tumor regression was observed following only one dose of rMV-EGFP-SLAMblind (Figure 4B). These results demonstrate that rMV-SLAMblind has an anti-tumor effect *in vivo*.

To further examine whether rMV-SLAMblind can target the tumors of the lung, a lung metastasis model was used. Lung tumors were established by intravenous injection of NCI-H441/CMV-Luc cells, which stably express the luciferase gene, and tumor growth was subsequently monitored by detection of luminescence using IVIS. At day 44 post-transplantation, when tumors could be visualized clearly in the lungs, rMV-EGFP-SLAMblind was serially administered to animals intravenously. Mice were euthanized and the excised lungs were examined by IVIS and fluorescent microscopy. IVIS analysis revealed that tumor luminescence and fluorescence from the virus replication in the tumor cells were observed in the same area (data not shown). Analysis by microscopy revealed that multiple tumors had grown in the lungs. The number of tumors varied from 16 to 36 per lung. Fluorescence was detected in many tumors, and was dependent upon rMV-SLAMblind-EGFP administration (Figure 4C, 4D), indicating that rMV-SLAMblind had infected the cells of multiple tumors established at different locations within the lung.

## DISCUSSION

Virotherapy is expected to play an important role in next-generation cancer therapy, especially in the area of drug-resistant cancers [25]. Late stage NSCLCs are often resistant to chemotherapy and radiotherapy, and have a poor 5-year survival rate [26, 27]. In this study, we aimed to determine the suitability of rMV-SLAMblind as a tool for lung cancer virotherapy, and demonstrated that this recombinant virus can infect many NSCLC cell lines. Several NSCLC cell line have been reported to express Nectin-4 (examples include NCI-H358, NCI-H2170, NCI-H441 and Calu-3) [6, 12]. Nectin-4 was discovered as the third receptor for MV infection using a mutant of a wild-type MV strain (IC-B strain) in NCI-H358 and NCI-H441 cells. However, the potential anti-tumor effects of this virus were not evaluated in previous studies [5, 18]. Here, we have demonstrated that rMV-SLAMblind, which is an attenuated virus, has an anti-tumor effect against NSCLC *in vitro* and *in vivo*, and that rMV-SLAMblind can target scattered tumor masses in the lungs by intravenous administration. Furthermore, several mice that had been transplanted with NCI-H441/CMV-Luc cells grew tumors in other regions as well as in the lungs, and fluorescence from virus-infected cells could also be detected in these tumors (data not shown). These findings suggest that intravenous administration of rMV-SLAMblind could be effective as a lung cancer therapy, and also may target metastatic tumors as well as primary lesions. Because rMV-SLAMblind does not have an affinity for immune cells [19], rMV-SLAMblind likely reaches Nectin-4-expressing tumor cells by blood flow but not through the spread of the virus-infected immune cells.

Several viruses have been proposed for use in lung cancer therapy and the MV vaccine strain has previously been shown to infect lung cancer cells [28, 29]. The anti-tumor effect of the MV vaccine strain was demonstrated using a NSCLC cell line (A549), which does not express Nectin-4 [28, 29]. Our findings suggest that rMV-SLAMblind that targets Nectin-4 is effective for lung cancer treatment. In addition, while the MV vaccine strain required repeated administration to suppress tumor growth *in vivo* [28, 29], one dose of recombinant virus led to tumor regression in our study. Because the anti-tumor effect of rMV-SLAMblind was shown to be more potent than that of the MV vaccine strain in a mouse xenograft model of breast cancer [19], a derivative of wild-type MV may be more effective than the MV vaccine strain for lung cancer treatment. Furthermore, a recent study reported that a MV vaccine strain infects NSCLC through CD46 but not Nectin-4 [30]. We propose the development of rMV-SLAMblind as a tool for virotherapy distinct from the use of MV vaccine strains.

The impediment by anti-MV neutralization antibody to the delivery of rMV-SLAMblind should be considered before use of this virus in clinical cases, because most of patients have preexisting anti-MV antibody resulting from measles vaccination. However, it was reported that mice xenografted with myeloma cells and treated with a MV vaccine strain survived longer than untreated mice even when anti-MV antibody was present in the mice [31]. We also performed a preliminary experiment to examine the effect of anti-MV neutralizing antibodies on the efficacy of rMV-SLAMblind. We inoculated five xenograft mice with human sera possessing neutralizing antibody to MV (×32) two days before the inoculation of rMV-Luc-SLAMblind. Luciferase activity was observed in all five positive control mice that had not been inoculated with human sera as well as in three of the five mice that had been inoculated (data not shown). These results suggest that the virus can reach and infect the target tumor cells even in the presence of anti-MV neutralizing antibodies. Once the virus infects and replicates in tumor cells, tumor antigen cross-presentation will likely be induced, as reported for virotherapy with the MV vaccine strain [32]. The combined use of rMV-SLAMblind with other drugs, such as dichloroacetate or inducer of mitophagy, to increase the effectiveness of virotherapy may be efficient [33, 34].

Other virus species have also been studied to determine their suitability for cancer virotherapy. There are reports that adenovirus and reovirus are effective against NSCLC [35, 36]. Different therapeutic strategies are often combined during cancer treatment to overcome the problem of resistance. Similar issues are likely to arise in virotherapy, such as resistance caused by mutations in cancer cells, and the immune response of the patient. Therefore, it is important to prepare many different virus species that are applicable for the treatment of cancer. rMV-SLAMblind will extend the repertoire of recombinant viral tools available for the virotherapy of lung cancer.

## MATERIALS AND METHODS

### Cell lines and culture

NCI-H358, NCI-H1666, and NCI-H2170 were purchased from the American Type Culture Collection (Rockville, MD, USA). NCI-H441/CMV-Luc was purchased from the National Institute of Biomedical Innovation (Osaka, Japan). Cells were maintained in culture according to suppliers' protocols. ABC-1, Calu-3, A431, PC14, NCI-H441, VMRC-LCD, RERF-LC-MS, NCI-H522, SKLU1, RERF-LC-OK, SBC-1, SBC-2, SBC-3, SBC-5, NCI-H69, N417, Lu139, and Lu134A were cultured as previously described [23]. Briefly, ABC-1, Calu-3, RERF-LC-MS, RERF-LC-OK, VMRC-LCD, SK-LU-1, SBC1, SBC2, SBC3, and SBC5 were maintained in minimum essential medium (MEM) supplemented with 10% fetal calf serum (FCS), 1 mM sodium pyruvate, and non-essential amino acids. NCI-H441, NCI-H522, PC-14, NCI-H69, N417, Lu134A, and Lu139 were maintained in RPMI 1640 medium supplemented with 10% FCS. MCF7 human breast cancer cells (obtained from the Cell Resource Center for the Biomedical Research Institute of Development, Aging and Cancer, Tohoku University, Miyagi, Japan) were cultured as previously described [19].

### Viruses

The generation of rMV-SLAMblind encoding green fluorescent protein (rMV-EGFP-SLAMblind) has been described previously [19]. Virus particles were harvested as follows: MCF7 cells were inoculated with the virus. Infected cells were then harvested with the culture supernatant and subjected to three cycles of freeze thawing and three rounds of sonication (8 s each) to release virus particle. The supernatant containing virus was then collected after centrifugation at 1940 × g for 10 min at 4°C. To obtain a high-titer virus for use in *in vivo* studies, viral particles were concentrated. Briefly, 200 mL of virus suspension was centrifuged at 19,000 rpm for 2 h at 4°C using a Beckman SW19 rotor (Beckman Coulter, Inc., Brea, CA, USA). The pellet was collected and re-suspended in approximately 1 mL medium, and stored at −80°C in aliquots. Virus titer (expressed as TCID_50_/mL) was determined using MCF7 cells as described previously [19].

### Virus infection of lung cancer cells

Cells were cultured in a 24-well plate and inoculated with rMV-EGFP-SLAMblind at a moi of 0.1 or 2. Infection of the virus was observed at various times after inoculation under a confocal microscope (FV1000; Olympus, Tokyo, Japan).

### Flow cytometry

Cells were washed with phosphate buffered saline (PBS) and trypsinized in 0.025% trypsin/0.24 mM ethylenediaminetetraacetic acid (EDTA). They were pelleted by centrifugation at 3,300 × g for 1 min, and then were resuspended in Hank's balanced salt solution (HBSS; Life Technologies) containing 2% FCS and incubated on ice for 30 min with anti-human Nectin-4 monoclonal antibody (Clone 337516, R&D Systems, Minneapolis, MN, USA), anti-human SLAM antibody [A12 (7D4); BioLegend, San Diego, CA, USA], and anti-CD46 antibody (M177; HyCult Biotech, Uden, The Netherlands). Next, the cells were washed in PBS containing 2% FCS, and incubated on ice for a further 30 min with Alexa 488-conjugated anti-mouse IgG (Life Technologies). Finally, the cells were washed with PBS containing 2% FCS, and the intensity of fluorescence was measured by a FACSCalibur (BD Biosciences, San Jose, CA, USA). To derive the relative level of Nectin-4 expression, the mean fluorescent intensity (MFI) of cells stained with anti-Nectin-4 antibody was calculated using Flowjo software ver 9.7.5 (TreeStar, San Carlos, CA).

### Cell proliferation assay

Cells were washed with PBS and trypsinized in 0.025% trypsin/0.24 mM EDTA. Cells (1.25 × 10^5^) were pelleted by centrifugation at 220 × g for 3 min. The cells were then resuspended in 300 μL of culture medium or virus inoculum containing rMV-EGFP-SLAMblind at a moi of 1. The cells were incubated at 37°C for 1 h, pelleted by centrifugation at 220 × g for 3 min to remove the inoculums, and resuspended in 5 mL of culture medium containing 2% FCS. The cells were then seeded into 96-well plates at a density of 5 × 10^3^ cells per well in 200 μL media and cultured at 37°C. Cell viability was determined using the WST-1 Cell Proliferation kit (Takara BIO INC., Otsu, Shiga, Japan) at 1, 3, 5, and 7 days post-infection (dpi), according to the manufacturer's protocol. The viability of infected cells was expressed as a percentage of the mean of quadruplicate absorbance values obtained for the infected cell population divided by that of the uninfected cell population.

### Xenograft model

Animal experiments were approved by the Experimental Animal Committee of The University of Tokyo. Five-week-old female severe combined immune deficiency (SCID) mice were purchased from CLEA Japan (Tokyo, Japan). A cell suspension of 1 × 10^8^/mL NCI-H441 in HBSS containing 2% FCS was mixed with an equal volume of Matrigel (BD GF Reduced, BD Biosciences), and 100 μL of this suspension was then injected subcutaneously into mice (5 × 10^6^ cells/mouse). The tumor volume was calculated as (width^2^ × length)/2. Following tumor growth, 10^6^ TCID_50_ of rMV-EGFP-SLAMblind was administered intratumorally into mice either 5 days or 13 days after implantation. The Wilcoxon-log-rank test was performed for the analysis of differences in tumor volume using JMP software (JMP Pro 10.0.2, SAS Institute Inc., Cary, NC, USA).

To establish lung tumors, NCI-H441/CMV-Luc cells (1 × 10^6^ cells in 100 μL) were introduced into the mice intravenously. To visualize tumor cells, mice were injected with 200 μL of D-luciferin (10 mg/ml; Gold Biotechnology, Inc., St. Louis, MO, USA) subcutaneously. Luminescence was measured using the Xenogen IVIS Imaging System 100 (IVIS; Xenogen/Caliper Life Sciences, Alameda, CA, USA) to monitor the tumor growth. A 15-cm field of view with 8 × 8 binning and an exposure time of 1 min, were used as imaging parameters. At day 44 post-transplantation, 10^6^ TCID_50_ of rMV-EGFP-SLAMblind in a total volume of 100 μL was administered intravenously into mice. Subsequently, 5 × 10^6^ TCID_50_ of rMV-EGFP-SLAMblind in 250 μL was administered intravenously into mice at 14, 41, and 48 dpi. Mice were then euthanized and the lungs were examined by fluorescent microscopy (MVX10; Olympus).
